# Pharmacological Interventions in Paraphilic Disorders: Systematic Review and Insights

**DOI:** 10.3390/jcm13061524

**Published:** 2024-03-07

**Authors:** Chiara Culos, Massimo Di Grazia, Paolo Meneguzzo

**Affiliations:** 1 Department of General Psychology, University of Padova, 35128 Padova, Italy; 2 Infermi Hospital, AUSL Romagna, 35128 Rimini, Italy; 3 Department of Neuroscience, University of Padova, 35128 Padova, Italy; 4 Padova Neuroscience Center, University of Padova, 35128 Padova, Italy

**Keywords:** paraphilic disorders, pharmacotherapy, serotonergic system, side effects

## Abstract

(1) **Background**: Paraphilic disorders, marked by intense sexual fantasies and behaviors, present formidable challenges. This review addresses concerns fueled by scandals and child abuse. Emphasizing paraphilias’ complexity, it systematically reviews the pharmacotherapy literature, aiming to enhance understanding and guide future research. (2) **Methods**: A comprehensive search from 1990 to 2023 across major databases identified 28 relevant English-language studies. Inclusion criteria focused on adult pharmacotherapy for paraphilias, and results were evaluated using the Newcastle–Ottawa Scale. (3) **Results**: Synthesizing data from selected studies, diverse treatments such as SSRIs and antiandrogens were analyzed, revealing variable effectiveness and side effect profiles. Poor quality of the current literature has been reported. (4) **Conclusions**: Highlighting the pivotal role of the serotonergic system, this review underscores the efficacy of SSRIs and androgen deprivation therapy. GnRH analog-associated side effects and the importance of a combined assessment approach are discussed. Critical insights contribute to understanding and ethical considerations in paraphilic disorders.

## 1. Introduction

A paraphilic disorder is defined by the DSM-5 as the persistent presence of intense sexually arousing fantasies, urges, or behaviors, typically involving nonhuman objects, self or partner suffering or humiliation, and nonconsenting individuals, with the potential to cause harm [1].

Recent years have witnessed a growing concern regarding paraphilia and its treatment, spurred by widely publicized scandals involving the clergy, politicians, and notorious cases of child abuse and murder. Alarming statistics reveal that approximately half a million children fall victim to sexual molestation in the United States annually, with similarly disturbing proportions reported in France, Germany, Belgium, the UK, Canada, and Denmark [2,3,4]. Paraphilias often coexist with other psychological conditions, leading to comorbidity. Research suggests that individuals with paraphilias may also exhibit symptoms of other paraphilias simultaneously [5]. For instance, some individuals diagnosed with pedophilia may also engage in exhibitionistic or voyeuristic behaviors. Additionally, certain forms of hypersexuality can be associated with exhibitionistic, voyeuristic, and other paraphilic behaviors. Moreover, individuals with paraphilic disorders frequently experience comorbid general psychiatric disorders [6]. Mood disorders, social anxiety disorder, autistic spectrum disorders, and ADHD are among the conditions commonly found alongside paraphilic disorders in sexual offenders.

Despite societal perceptions that some paraphilias, such as pedophilia, are primarily criminal acts, extensive efforts have been made to refine paraphilia definitions and develop comprehensive treatments [7,8,9]. It is crucial to understand that paraphilias are intricate and heterogeneous disorders, displaying characteristics reminiscent of addictive, impulsive, or obsessive compulsive disorders, and often manifesting as patterns of hypersexuality [6,8]. Typically, individuals with paraphilic tendencies are referred to psychiatrists by legal authorities, with rape (involving a child or otherwise) and exhibitionism being predominant reasons for consultation. Importantly, not all sex offenders meet the criteria for paraphilia, and paraphilia is not synonymous with being a sex offender; for instance, not all rapists necessarily exhibit paraphilic tendencies [10].

Recently, a change in the treatment paradigm has been proposed, showing the differences between managing and treating paraphilic disorders [11,12]. While managing paraphilic disorders focuses on symptom control, harm reduction, and improving daily functioning, treating paraphilic disorders involves addressing underlying issues, facilitating symptom reduction or remission, and promoting long-term psychological well-being and recovery. Both approaches play crucial roles in supporting individuals with paraphilic disorders, often as part of a multifaceted treatment plan tailored to meet their specific needs and goals. In addition to psychotherapeutic interventions, pharmacotherapy emerges as a pivotal approach in sexual offender therapy [8,13]. Several reviews of the literature have suggested that the combined use of pharmacotherapy and psychotherapy, as well as outpatient treatment compared with inpatient treatment, demonstrates superior efficacy in reducing recidivism [12,14,15,16].

The primary goal of this comprehensive review is to delve into the existing literature on paraphilic disorders, with a specific focus on evaluating various treatments and elucidating reported side effects. By synthesizing current knowledge on treatment modalities, their efficacy, and associated adverse effects, we aim to contribute to a nuanced understanding of the complexities surrounding this sensitive area of mental health. Our objective is to provide valuable insights that may guide future research endeavors and contribute to the ongoing development of effective and ethically sound interventions for individuals grappling with paraphilic disorders.

## 2. Materials and Methods

### 2.1. Literature Search and Inclusion Criteria

**Figure 1:**
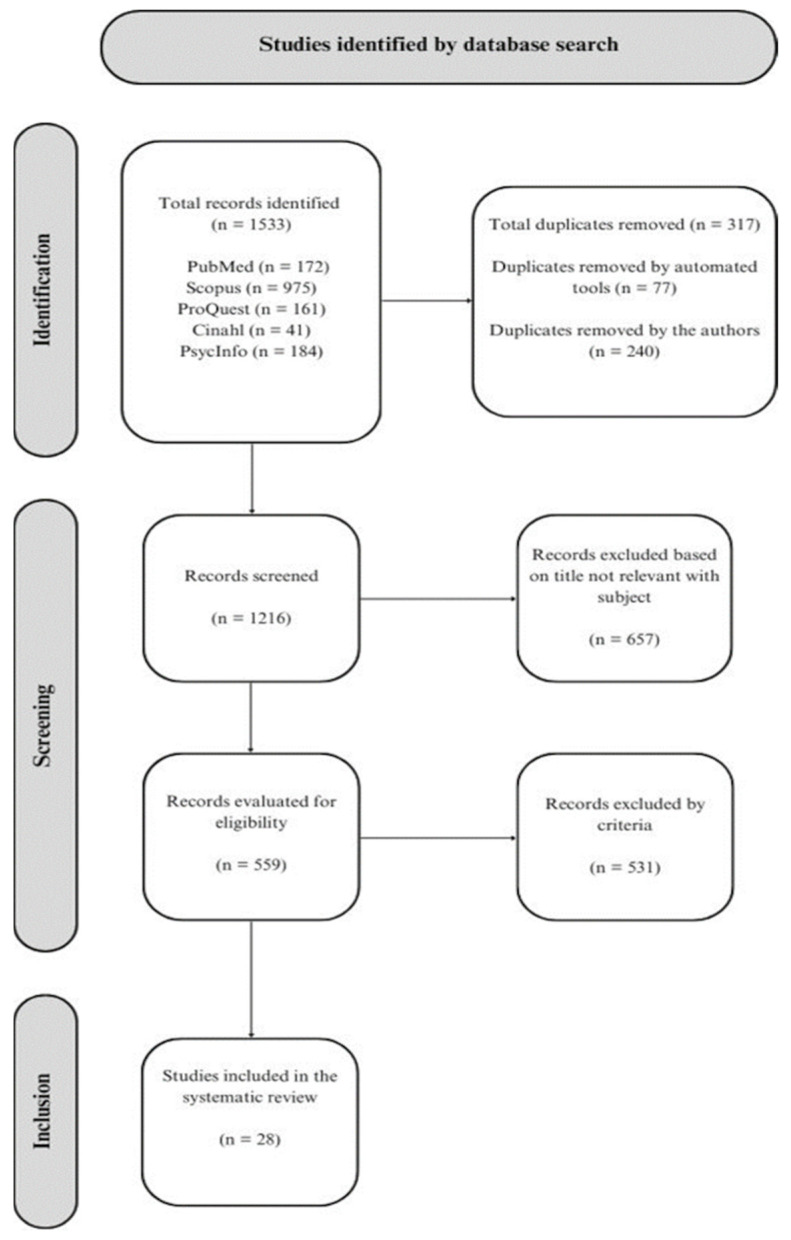
The PRISMA flowchart of the revision.

We conducted a comprehensive search for empirical studies published exclusively in English from 1990 to 2023. The last search was performed on 28 July 2023, using five search engines: PubMed, Scopus, CINAHL, ProQuest, and PsycINFO. The keywords used were paraphilia, paraphilic disorder, pharmacotherapy, cyproterone acetate, medroxyprogesterone acetate, gonadotropin-releasing hormone analog, gonadotropin-releasing hormone agonist, selective serotonin reuptake inhibitor, naltrexone, fluoxetine, sertraline, fluvoxamine, paroxetine, citalopram, antiandrogen, tricyclic antidepressant, triptorelin, leuprorelin, leuprolide acetate, and androgen deprivation. A total of 1533 papers were identified; see Figure 1 for the PRISMA flowchart. Our search criteria were deliberately broad for clinical phenomena and more restricted for diagnosis and patient characteristics. This systematic review was not registered.

Studies concerning pharmacotherapy, used either as monotherapy or in combination with psychotherapy or other interventions, conducted on adult individuals (age 18 years and older) of any gender diagnosed with paraphilia, were included. Open-label, single- or double-blinded studies, and observational or case–control studies were included, with no restrictions regarding clinical setting. Studies reporting paraphilias as a side effect of other medications or in comorbidity with other disorders or diseases were excluded. In the case of sex offenders, studies were included only if those individuals had formal diagnoses of paraphilia. Studies regarding compulsive sexual behavior (or hypersexuality) were excluded because it is not yet a disorder classified in the DSM-5 and therefore lacks precise diagnostic criteria; however, they were considered if the patient had other paraphilias. Hypersexuality and compulsive sexual behavior represent complex phenomena within the realm of sexual health and psychiatric disorders. While often used interchangeably, hypersexuality denotes an excessive preoccupation with sexual thoughts, desires, or behaviors, while compulsive sexual behavior involves persistent engagement in sexual activities despite negative consequences [17,18]. Despite its clinical significance, hypersexuality is not classified as a distinct disorder in the DSM-5 due to the lack of consensus on diagnostic criteria and classification. The exclusion of hypersexuality reflects ongoing debates within the psychiatric community regarding its conceptualization and classification. Pharmacological treatments for hypersexuality remain an area of ongoing research, with medications targeting neurotransmitter systems implicated in regulating sexual behavior showing promise in reducing impulsivity and compulsivity. While hypersexuality may be mentioned in articles discussing specific paraphilias or related psychiatric conditions, its distinction from other sexual disorders underscores the need for further research and clinical understanding to inform effective assessment and treatment approaches.

Eligibility criteria were established through consensus among two authors (CC, PM). Any discrepancies were resolved through discussion with a third. A wide-ranging set of criteria determined the clinical phenomena, while a more focused set guided the selection based on diagnosis and patient characteristics.

### 2.2. Quality Assessment

The Newcastle–Ottawa Scale (NOS) assesses the quality of case–control and longitudinal studies, encompassing three domains: (a) participant selection, (b) comparability between cases and controls, and (c) accuracy of outcome evaluation [19]. These domains include eight specific items, with slight variations for case–control and longitudinal studies. A study can score a maximum of 9 points, and those with less than 5 points are deemed at high risk of bias.

## 3. Results

### 3.1. Included Studies

After applying the eligibility criteria, 28 papers, comprising a total of 379 patients, were included in the review. Treatment modalities encompassed SSRIs, steroid antiandrogens, and GnRH agonists or analogs. Additionally, studies exploring emerging drug categories, such as anxiolytics and antidepressants, currently under investigation as potential new pharmacotherapies, were incorporated. A detailed presentation of participant information, diagnoses, therapies employed, obtained results, and any observed side effects can be found in Table 1.

### 3.2. Quality Assessment

Using the NOS, it appeared that most of the studies were at high risk of bias, mainly due to the lack of a control group, small numbers of participants, and the use of self-reports. Only three of the reported studies ([28,36,43]) had NOS scores greater than five. The scores are reported in Table 2.

### 3.3. Evaluation of Effectiveness

#### 3.3.1. Selective Serotonin Reuptake Inhibitors (SSRIs)

Paroxetine was evaluated in treating voyeurism and exhibitionism [20]. In the first case, the patient initially received fluoxetine and paroxetine, both proving effective. Switching to an evening dose of paroxetine further improved impulse control, and increasing the dose from 10 mg to 20 mg significantly reduced deviant thoughts, voyeuristic behaviors, and pornography use. Similar positive outcomes were observed in the second case, with an additional dose increase to 30 mg for persistent deviant thoughts.

Sertraline was explored on a patient with pedophilia, resulting in a decrease in pedophilic thoughts and improved impulse control without side effects [21]. However, this was not the case in another patient with exhibitionism, pedophilia, and prior alcohol abuse, where sertraline, topiramate, and intramuscular haloperidol were not effective until medroxyprogesterone acetate was added [22].

Fluoxetine was tested for voyeurism [23], leading to the suppression of deviant thoughts after three months at a daily dose of 60 mg. In a case of exhibitionism resistant to psychodynamic and behavioral therapy, fluoxetine at 40 mg was effective, with sustained results at six-month follow-up [25]. Conversely, a study [26] reported a masochism case where initial treatment with leuprolide acetate reduced sexual arousal but discontinuation led to a lack of improvement with fluoxetine. The patient experienced decreased fantasies during combined fluoxetine and psychotherapy, with the most significant improvement seen through aversive techniques when fluoxetine was not administered.

#### 3.3.2. Steroidal Antiandrogens

In a retrospective observational study [27], the effectiveness of combining cyproterone acetate (CPA) and GnRH analogs with psychotherapy was examined in 111 patients with pedophilia. They analyzed the progression of the disorder and cognitive changes, such as empathy and self-efficacy. Fifteen patients received both psychotherapy and pharmacotherapy, while 96 underwent psychotherapy alone. The results indicated that patients under pharmacotherapy experienced a reduction in deviant behavior and an increase in perceived self-efficacy.

Similarly, a double-blind study [28] conducted on 19 patients with various paraphilias explored hormonal and psychological effects in three therapy phases. Hormone levels showed no significant differences between baseline and placebo phases, while changes were observed in testosterone and follicle-stimulating hormone between baseline and placebo phases compared to the active phase. Psychologically, there was a decrease in the total score on the Brief Psychiatric Rating Scale (BPRS, [49]).

A study [29] compared the effects of CPA and triptorelin, along with cognitive behavioral therapy, in 12 patients with pedophilia. Nine participants reported decreased sexual activity and fantasies, emphasizing the benefits of antiandrogens in combination with psychotherapy. CPA showed some reluctance and side effects, while triptorelin resulted in weight gain and bone mass loss.

Differently, CPA and leuprolide acetate were compared in a patient with pedophilia over 54 months, finding a greater decrease in testosterone levels and phallometric tests with leuprolide acetate than with CPA [30].

Regarding medroxyprogesterone acetate (MPA), the first case in 1966 showed a radical decrease in testosterone, leading to the cessation of deviant urges. Subsequent studies, however, presented mixed results. Some patients experienced complete remission, while others had inconsistent responses, with side effects like glaucoma and migraine leading to discontinuation.

Despite the efficacy in reducing deviant fantasies, severe side effects such as gynecomastia and obesity have been reported in some cases. The correlation between MPA, group therapy, and individual psychotherapy in preventing recidivism was also studied, with varying results and a potential predictive factor being the initial plasma testosterone concentration.

In summary, while there is evidence of efficacy in using antiandrogens like CPA, leuprolide acetate, and MPA in conjunction with psychotherapy for paraphilias, the variability in responses and potential side effects emphasize the need for careful monitoring and individualized treatment plans.

#### 3.3.3. GnRH Agonist and Analogue

The effectiveness of leuprolide acetate was evaluated in sex offenders diagnosed with paraphilia [36]. The study involved administering questionnaires such as the Minnesota Multiphasic Personality Inventory (MMPI), the Sex Addiction Screening Test (SAST), and Beck’s Depression Inventory (BDI), along with analyzing hormone levels. After the first leuprolide injection, patients received psychotherapy and monthly injections. The results, evident after one month, showed a reduction in sexual desire, changes in the MMPI masculinity/femininity scale, and a decrease in hormone levels. Some reported side effects, including eczema and nausea, emerged at the one-year follow-up, resolving with a consistent leuprolide dosage.

Promising results were reported with leuprolide acetate in a hypersexuality and exhibitionism case, noting reduced phallometry and hormone levels [37], confirmed after depoMPA and depoCPA failed to show improvement.

Functional magnetic resonance imaging (fMRI) was used to evaluate leuprolide acetate’s effects in a patient with homosexual pedophilia [39]. The study demonstrated a decrease in amygdala activation in response to erotic stimuli, aligning with reduced erotic valence reported by the patient. Similar results were confirmed by another study in a patient with pedophilia [40].

Osteoporosis, a common side effect, occurred in a patient treated with leuprolide acetate but improved with calcium and vitamin D supplementation [41]. The use of degarelix, a GnRH antagonist, in preventing child sexual abuse was explored, observing reduced risk scale scores after two weeks [42]. However, some patients in the experimental group experienced hepatobiliary enzyme increases and suicidal ideation.

Finally, triptorelin injections in combination with psychotherapy showed a drastic decrease in scores for sexual desire, activity, and fantasies [44]. Despite hormonal level decreases, some patients experienced side effects such as osteoporosis, hot flushes, and muscle tension. Moreover, the successful use of an LHRH agonist with flutamide was reported in treating severe exhibitionism, resulting in reduced testosterone, a complete remission of deviant activities, and decreased sexual fantasies, with hot flushes as the only side effect [45].

#### 3.3.4. Other Drugs

Regarding topiramate, this anticonvulsant was utilized in treating fetishism [46]. After unsuccessful psychotherapy, symptoms reduced, and the ability to control sexual impulses developed approximately one month into therapy, with no reported side effects.

In an interesting case report, bupropion was used to treat exhibitionism unresponsive to serotonergic antidepressants [47]. Initially, psychotherapy and escitalopram were ineffective. A subsequent administration of paroxetine and trazodone, despite improving sleep, did not reduce sexual fantasies even with increased dosage. The patient then underwent pharmacotherapy with sertraline in conjunction with psychotherapy, resulting in minimal impulse reduction. However, introducing bupropion led to a significant decrease in deviant fantasies and impulses within just one month.

Finally, a case of exhibitionism and obscene telephone calling treated with buspirone and psychotherapy was found [48]. Deviant behaviors ceased on the first day of pharmacotherapy, as confirmed by Y-BOCS scores, with the suppression of fantasies and deviant behaviors lasting over 30 months.

## 4. Discussion

Based on the reported studies, the pharmacotherapy of paraphilias remains diverse and uncertain. According to the pharmacological treatment guidelines [50], therapy is primarily based on the impact of drugs on serotonergic systems and reducing hormone levels, especially testosterone. Elevated testosterone levels, akin to castration levels, contribute to decreased sexuality and, in diagnosed cases, a reduction in deviant fantasies and behaviors, but with several side effects. Therefore, regular monitoring of testosterone, luteinizing hormone, and follicle-stimulating hormone levels is essential, serving both as a continuous evaluation of the patient’s well-being and an objective measure for potential dosage adjustments.

The aim of this review was to offer clinical insights for practitioners dealing with paraphilias. However, comprehensive clinical insights remain elusive. While SSRIs show promise with minimal reported side effects, the evidence is limited to six case reports involving seven individuals. Steroidal antiandrogens present the highest level of evidence, encompassing 142 patients, yet the main outcomes predominantly focus on behavioral improvements, including reduced deviant behaviors and enhanced impulse control. GnRH agonists or analogs similarly demonstrate decreased sexual fantasies and hormonal levels, alongside improved impulse control, albeit with notable side effects. Notably, only a few studies achieved a high-quality score in terms of bias reduction, predominantly in the steroidal and GnRH drug categories. Overall, the efficacy and side effect profiles of paraphilia treatments vary across medication classes. While certain medications exhibit promise in curbing deviant behaviors and impulses, the individualized nature of responses underscores the importance of careful monitoring and treatment planning. Furthermore, combining medication with psychotherapy appears to augment treatment outcomes. The treatment involves various drug classes, each yielding conflicting results due to differing measurement methods. Some studies rely on self-report questionnaires, such as the Sexual Desire Inventory and Hypersexual Behavior Inventory [43], revealing a decrease in sexual sphere scores post-drug administration. However, self-reports have limitations, with social desirability influencing patient responses [31]. Therefore, self-report questionnaires should only be employed in studies with objective, empirical feedback, such as phallometric testing or hormone level measurements. Phallometric testing is a psychological assessment utilized in forensic and clinical settings to measure sexual arousal patterns, especially in response to visual or auditory stimuli [51,52]. Despite a growing body of literature and raised doubts about its results, it is still considered a reliable measurement. The test assesses changes in penile blood volume or circumference as indicators of sexual arousal. Primarily, this test is employed in sex offender assessment and treatment contexts, as well as in research concerning sexual behavior and psychology.

At the neural level, structures implicated in paraphilias are discussed [40,53]. Pharmacotherapy studies reveal the impact of drugs on neuronal activation, with leuprorelin demonstrating decreased activation in the amygdala and subcortical areas [39,40]. Generally, SSRI drugs and androgen deprivation therapy prove the most effective, primarily reducing deviant fantasies with minimal side effects. In our review of the literature, this aspect appears to be supported by examining the minimal effect size reported (delayed ejaculation) in comparison to the reduction of dysfunctional symptomatology. SSRIs are recognized as viable drugs with a favorable effect-to-side-effect ratio, and the evaluation of the studies found in the paraphilic literature supports this [3].

The rationale behind androgen deprivation therapy involves a significant reduction in testosterone levels. Steroidal antiandrogens, such as medroxyprogesterone acetate and cyproterone acetate, effectively inhibit luteinizing hormone production, resulting in decreased testosterone. Despite achieving remission in fantasies and deviant arousal, these drugs often lead to side effects like muscle cramps, weight gain, migraine, edema, and gynecomastia [31,32,33]. GnRH analogs and agonists are also used, demonstrating a dramatic decrease in hormone levels but frequently causing osteoporosis. Therefore, physiological monitoring, along with calcium and vitamin D supplementation, is recommended.

Research explores alternative drug categories, such as antidepressants and anxiolytics, based on their impact on the serotonergic system. For instance, buspirone, an anxiolytic, inhibits serotonergic action and has shown efficacy in treating paraphilias [48]. Despite the positive results in drug efficacy studies, methodological shortcomings, including the lack of initial physiological assessments, absence of follow-up, reliance on self-reports, and low participant numbers, may impact results.

Physiological assessments are crucial for objective evaluations of physical and hormonal changes and side effects due to medication use. Relevant values include hormone levels (testosterone, luteinizing hormone, follicle-stimulating hormone), blood pressure, glucose levels, and bone density. Reported studies highlight side effects such as increased blood pressure, diabetes mellitus onset, and osteoporosis. Regarding bone density measurements following GnRH use, a study reports strong bone demineralization without an initial baseline value, emphasizing the need for precise measurement protocols [41].

The literature suggests employing neuroimaging techniques and physiological measures like phallometrics to monitor changes in brain structures and sexual behavior control. However, these techniques involve financial and temporal costs. Additionally, the importance of using professionally administered questionnaires over subjective patient assessments is emphasized, with evidence highlighting the role of placebos in patient-reported improvements not corresponding to physiological values [31].

Questionnaires addressing comorbidities like depression, anxiety, personality disorders, and obsessive compulsive disorder are crucial for selecting appropriate medications. For example, selective serotonin reuptake inhibitors may serve as both antidepressants and treatments for paraphilias with comorbid depression [21]. Research demonstrates that sertraline not only reduces paraphilic symptoms but also improves scores on depression and anxiety questionnaires [21].

Therefore, a combined approach utilizing self-report and physiological measures is recommended to mitigate external factors’ influence. A minimum three-month follow-up is advised to assess symptomatic consistency and potential long-term side effects. While moderate side effects were noted in [36], some treated patients discontinued therapeutic monitoring upon symptom remission, emphasizing the need for sustained follow-up. The importance of these factors in determining the correct drug dosage is underscored.

Specific limits have to be considered in the interpretation of the results. This review spans three decades (1990–2023) but is limited to English-language studies. The exclusion criteria include patients under 18, cases lacking objective paraphilia diagnosis, and cases of compulsive sexual behavior due to undefined diagnostic criteria. Finally, our review points out that the current literature has several flaws and limitations, requiring deeper studies and considerations. One aspect that requires special attention is the low quality of the studies presented in the current literature. This is partly because the NOS may not be suitable for studies employing different methodologies, even if other alternatives appear to be less effective.

It is essential to acknowledge a final consideration. The prevailing literature largely comprises case reports and case series, methodologies known for their limited reliability and generalizability of results. Consequently, future studies should strive to engage in consortiums or collaborative research endeavors to secure robust sample sizes and conduct longitudinal evaluations. Such an approach will facilitate the identification of reliable trends and offer more substantial insights.

## 5. Conclusions

Insights from reviewed studies underscore the critical role of the serotonergic system in the pharmacotherapy of paraphilias. Selective serotonin reuptake inhibitors and androgen deprivation therapy prove most effective in reducing paraphilic symptoms. However, noteworthy side effects, particularly with GnRH analogs and agonists, emphasize the importance of careful consideration.

Continued research is essential to explore alternative short-term treatments that alleviate symptoms without compromising the patient’s regular life. Ethical concerns surrounding androgen deprivation therapy warrant further investigation to optimize patient well-being and relational dynamics.

## Figures and Tables

**Table 1:** Study results of patients treated with pharmacotherapy.

Study	Samples and Diagnoses	Treatment	Duration of Treatment	Main Results	Side Effects
Selective serotonin reuptake inhibitors (SSRIs)					
Abouesh and Clayton (1999) [20]	two men with voyeuristic (A) and exhibitionistic (B) disorder	Paroxetine: A: 20 mg/day; B: 30 mg/day.	A: three months of treatment and then four months without B: six months of treatment and two months follow-up	Improved impulse control, decrease in fantasies and behaviors.	NR
Chow and Choy (2002) [21]	one woman sex offender with pedophilia	Sertraline (50 mg/day) + psychotherapy	12 months	Decrease in intensity and frequency of fantasies, increased impulse control.	NR
Baltieri and De Andrade (2009) [22]	one man with exhibitionism and pedophilia	(1) Sertraline (200 mg/day) + topiramate (200 mg/day) + haloperidol (300 mg/mo) + psychotherapy; (2) addition of MPA (300 mg/2 tmo).	four months of treatment and three months follow-up	(1) No decrease in fantasies; (2) absence of fantasies and deviant behaviors.	NR
Emmanuel et al. (1991) [23]	one man with voyeurism	Fluoxetine (60 mg/day)	3 months	Suppression of thoughts and deviant behaviors.	NR
Perilstein et al. (1991) [24]	three men	Fluoxetine	-	Improvement of impulse control	NR
Saleh and Berlin, (2004) [25]	one man with exhibitionism	Fluoxetine	6 months	Urge reduction and sexual desire.	Delayed ejaculation
Shiwach and Prosser (1998) [26]	one man with masochism	Fluoxetine (80 mg/day) + psychotherapy	42 weeks	Decreased fantasies and arousal.	NR
Steroidal antiandrogens					
Amelung et al. (2012) [27]	one hundred and eleven patients with pedophilia	(1) seven with CPA (300–600 mg/biweek) and seven with GnRHa + Psychotherapy; (2) ninety-six with psychotherapy. SSRI as an add-on if necessary.	3 months	(1) Decreased deviant behavior and increased self-efficacy.	NR
Bradford et al. (1993) [28]	nineteen patients with multiple paraphilias (1) + placebo (2)	(1) CPA (50–200 mg/day); (2) placebo (50–200 mg/day).	8 months	Decreased TS, FSH, LH, decreased BPRS scores.	Without any significant variations
Boons et al. (2020) [29]	twelve sex offenders with pedophilia	(1) CPA + CBT; (2) triptorelin + CBT.	-	Decrease in fantasies, urges, and behaviors.	(1) Gastric problems, reduced blood pressure; (2) weight gain, loss of bone mass.
Cooper and Cernovsky (1994) [30]	one man with pedophilia	(1) CPA (100–200 mg/day); (2) leuprolide acetate (7.5 mg/mo).	38 months	(1) Decrease in values; (2) suppressed TS levels, arousal, and self-report measures.	NR
Kiersch (1990) [31]	eight men with pedophilia	MPA (DepoProvera) (100–400 mg/week) + Saline (100–400 mg/week)	64 weeks	Remission fantasies, arousal with nondeviant stimuli. Decreased fantasies and frequency of masturbation with MPA. Decreased fantasies with saline. Decrease with saline, increase with MPA.	Glaucoma Migraine
Kravitz et al. (1995) [32]	twenty-nine men with various paraphilias	MPA (300 mg/week) + Group therapy MPA	6 months	Suppression of fantasies and deviant activities, increased ability to control impulses.	Muscle cramps, weight gain, migraine, fatigue, lethargy, drowsiness, depression, anxiety, pulmonary embolism.
Krueger et al. (2006) [33]	one man with pedophilia	MPA (300 mg/day)	4 years	Reduced sexual impulses.	Gynecomastia, obesity, adrenal insufficiency, Cushing’s syndrome.
Lehne and Money (2000) [34]	one man with multiple paraphilias	MPA	forty years’ follow-up	Decrease in TS levels, cessation of deviant urges.	Erectile dysfunction, weight gain, fatigue.
Meyer et al. (1992) [35]	forty sex offenders with various paraphilias	MPA (400 mg/week) + psychotherapy + Group therapy	from 6 months to 12 years	Eighteen percent reiteration of abuse with MPA, 35% reiteration after termination, 58% reiteration without MPA.	Weight gain, migraine headaches, cramps, increased blood pressure, diabetes mellitus.
GnRH agonists or analogs					
Choi et al. (2018) [36]	seven sex offenders with various paraphilias	Leuprolide acetate + psychotherapy	12 months	Decreased sexual fantasies, sexual interest, decreased scores on the questionnaires.	Feminization, fatigue, hot flushes.
Saleh (2005) [37]	one patient with hypersexuality and exhibitionism	Leuprolide acetate (7.5 mg/mo) + psychotherapy	5 months	Decreased urge and sexual drive, decreased LH, FSH, TS levels.	NR
Dickey (1992) [38]	one man with multiple paraphilias	Leuprolide acetate	32 months	Decreased TS levels, LH, decreased frequency of masturbation and cessation of deviant behavior.	NR
Habermeyer et al. (2011) [39]	one man with homosexual pedophilia	Leuprorelin (11.25 mg/3 mo)	-	Decreased TS levels, decreased activation of the amygdala.	NR
Schiffer et al. (2009) [40]	one man with pedophilia	Leuprorelin (11.25 mg/3 mo)	9 months	Decrease in processing of visual stimuli in subcortical areas.	NR
Dickey (2002) [41]	one man with multiple paraphilias	Leuprolide acetate	10 years	Decreased TS levels, increased ability to control.	Osteoporosis
Landgren et al. (2020) [42]	fifty-two men with pedophilia	(1) degarelix (120 mg); (2) placebo.	2 weeks	Decreased scores on scale of risk.	(1) Hepatobiliary enzyme increase, suicidal ideation; (2) NR.
Landgren et al. (2022) [43]	52 men with pedophilia	(1) degarelix (120 mg) [25]; (2) placebo [25].	10 weeks	Reduced SDI, HBI, decreased deviant interest.	NR
Rösler and Witton (1998) [44]	thirty patients with severe paraphilias	Triptorelin (3.75 mg/mo) + psychotherapy	8–42 months	Decreased questionnaire scores, decreased hormone levels, decreased fantasies.	Osteoporosis, hot flushes, muscle tension, erectile dysfunction.
Rousseau et al. (1990) [45]	one patient with exhibitionism	Flutamide + LHRH agonist	52 weeks	TS decrease, DHT, remission of deviant activities, decrease in sexual fantasies.	Hot flushes
Other drugs					
Shiah et al. (2006) [46]	one man with fetishism	Topiramate (200 mg/day)	6 months	Reduction in symptoms, development of ability to control.	NR
Vayisoglu (2023) [47]	one patient with exhibitionism	Bupropion (150 mg/day)	6 months	Reduction of deviant fantasies and impulses.	NR
Pearson et al. (1992) [48]	one patient with exhibitionism and telephone scatology.	Buspirone (25 mg/day) + psychotherapy	30 months	Termination of deviant behaviors, suppression of deviant fantasies.	NR

Between brackets are reported the number of participants exposed or that reported the symptoms. BPRS: Brief Psychiatric Rating Scale; NR: not reported; day: daily dose; mo: monthly dose; 2 tmo: dose administered twice a month; week: weekly dose; biweek: biweekly dose; SDI: Sexual Desire Inventory; HBI: Hypersexual Behavior Inventory; TS: testosterone; MPA: medroxyprogesterone acetate.

**Table 2:** Scores of Newcastle–Ottawa Scale (NOS).

Paper	Selection				Comparability	Exposure			Tot
Abouesh and Clayton (1999) [20]	1b	2b	-	-	-	1d	-	-	0
Chow and Choy (2002) [21]	1b	2b	-	-	-	1d	-	-	0
Baltieri and De Andrade (2009) [22]	1a	2b	-	-	-	1a	-	-	2
Emmanuel et al. (1991) [23]	1c	2b	-	-	-	1d	-	-	0
Perilstein et al. (1991) [24]	1a	2b	-	-	-	1d	-	-	1
Shiwach and Prosser (1998) [26]	1a	2b	-	-	-	1d	-	-	1
Dickey (1992) [38]	1b	2b	-	-	-	1d	-	-	0
Amelung et al. (2012) [27]	1a	2a	3b	4b	1a	1d	2a	3b	4
Bradford et al. (1993) [28]	1a	2a	3a	4b	1a	1a	2a	3b	6
Boons et al. (2020) [29]	1a	2a	3b	4b	1a	1d	2a	3b	4
Cooper and Cernovsky (1994) [30]	1b	2b	-	-	-	1d	-	-	0
Kiersch (1990) [31]	1c	2b	-	-	-	1d	-	-	0
Kravitz et al. (1995) [32]	1a	2b	-	-	-	1d	-	-	1
Krueger et al. (2006) [33]	1c	2b	-	-	-	1d	-	-	0
Lehne and Money (2000) [34]	1b	2b	-	-	-	1d	-	-	0
Meyer et al. (1992) [35]	1a	2a	3a	4b	1a	1d	2b	3b	4
Choi et al. (2018) [36]	1a	2a	3a	4b	1a	1a	2a	3b	6
Dickey (2002) [41]	1b	2b	-	-	-	1d	-	-	0
Habermeyer (2012) [39]	1a	2b	-	-	-	1a	-	-	2
Landgren et al. (2020) [42]	1b	2b	3a	4b	1a	1b	2a	3b	4
Landgren et al. (2022) [43]	1a	2a	3a	4b	1a	1d	2a	3b	5
Saleh (2005) [37]	1a	2b	-	-	-	1d	-	-	1
Schiffer et al. (2009) [40]	1c	2b	-	-	-	1d	-	-	0
Rousseau et al. (1990) [45]	1a	2b	-	-	-	1d	-	-	1
Rösler and Witztum (1998) [44]	1a	2a	-	-	-	1a	-	-	3
Shiah et al. (2006) [46]	1a	2b	-	-	-	1d	-	-	1
Vayisoglu (2023) [47]	1b	2b	-	-	-	1d	-	-	0
Pearson et al. (1992) [48]	1a	2b	-	-	-	1d	-	-	1

## Data Availability

Not applicable.

## References

[1] Beech A.R., Miner M.H., Thornton D. (2016). Paraphilias in the DSM-5. Annu. Rev. Clin. Psychol..

[2] Krueger R.B., Kaplan M.S. (2001). The paraphilic and hypersexual disorders: An overview. J. Psychiatr. Pract..

[3] Turner D., Petermann J., Harrison K., Krueger R., Briken P. (2017). Pharmacological treatment of patients with paraphilic disorders and risk of sexual offending: An international perspective. World J. Biol. Psychiatry.

[4] Walton M.T., Cantor J.M., Bhullar N., Lykins A.D. (2017). Hypersexuality: A critical review and introduction to the “sexhavior cycle”. Arch. Sex. Behav..

[5] Tozdan S., Briken P., Lew-Starowicz M., Giraldi A., Krüger T.H.C. (2021). Paraphilias: Diagnostics, Comorbidities, and Treatment. Psychiatry and Sexual Medicine: A Comprehensive Guide for Clinical Practitioners.

[6] Kafka M. (2012). Axis I Psychiatric Disorders, Paraphilic Sexual Offending and Implications for Pharmacological Treatment. Isr. J. Psychiatry Relat. Sci..

[7] Caldeano A.R., Nunes J., da Costa P. (2016). Paraphilic disorder in the 21st century. Eur. Psychiatry.

[8] Yakeley J. (2018). Psychoanalytic perspectives on paraphilias and perversions. Eur. J. Psychother. Couns..

[9] First M.B. (2014). DSM-5 and paraphilic disorders. J. Am. Acad. Psychiatry Law. Online.

[10] Di Lorenzo G., Gorea F., Longo L., Ribolsi M. (2018). Paraphilia and paraphilic disorders. Sexual Dysfunctions in Mentally Ill Patients.

[11] Baez-Sierra D., Balgobin C., Wise T.N. (2016). Treatment of Paraphilic Disorders. Practical Guide to Paraphilia and Paraphilic Disorders.

[12] Holoyda B.J., Kellaher D.C. (2016). The biological treatment of paraphilic disorders: An updated review. Curr. Psychiatry Rep..

[13] Krueger R.B., Kaplan M.S. (2002). Behavioral and psychopharmacological treatment of the paraphilic and hypersexual disorders. J. Psychiatr. Pract..

[14] Greenberg D.M., Bradford J.M.W. (1997). Treatment of the paraphilic disorders: A review of the role of the selective serotonin reuptake inhibitors. Sex. Abus..

[15] Turner D., Briken P. (2018). Treatment of Paraphilic Disorders in Sexual Offenders or Men with a Risk of Sexual Offending with Luteinizing Hormone-Releasing Hormone Agonists: An Updated Systematic Review. J. Sex. Med..

[16] Winder B., Fedoroff J.P., Grubin D., Klapilova K., Kamenskov M., Tucker D., Basinskaya I.A., Vvedensky G.E. (2019). The pharmacologic treatment of problematic sexual interests, paraphilic disorders and sexual preoccupation in adult men who have committed a sexual offence. Int. Rev. Psychiatry.

[17] Landgren V., Savard J., Dhejne C., Jokinen J., Arver S., Seto M.C., Rahm C. (2022). Pharmacological Treatment for Pedophilic Disorder and Compulsive Sexual Behavior Disorder: A Review. Drugs.

[18] Gola M., Lewczuk K., Potenza M.N., Kingston D.A., Grubbs J.B., Stark R., Reid R.C. (2022). What should be included in the criteria for compulsive sexual behavior disorder?. J. Behav. Addict..

[19] Wells G., Shea B., O’Connell D., Peterson J., Welch V., Losos M., Tugwell P. (2015). The Newcastle-Ottawa Scale (NOS) for assessing the quality of nonrandomised studies in meta-analyses.

[20] Abouesh A., Clayton A. (1999). Compulsive Voyeurism and Exhibitionism: A Clinical Response to Paroxetine. Arch. Sex. Behav..

[21] Chow E.W.C., Choy A.L. (2002). Clinical Case Report Series 1 Clinical Characteristics and Treatment Response to SSRI in a Female Pedophile. Arch. Sex. Behav..

[22] Baltieri D.A., De Andrade A. (2009). Treatment of paraphilic sexual offenders in brazil: Issues and controversies. Int. J. Forensic Ment. Health.

[23] Emmanuel N.P., Lydiard R.B., Ballenger J.C. (1991). Fluoxetine treatment of voyeurism. Am. J. Psychiatry.

[24] Perilstein R.D., Lipper S., Friedman L.J. (1991). Three cases of paraphilias responsive to fluoxetine treatment. J. Clin. Psychiatry.

[25] Saleh F.M., Berlin F.S. (2004). Sex hormones, neurotransmitters, and psychopharmacological treatments in men with paraphilic disorders. J. Child Sex. Abus..

[26] Shiwach R.S., Prosser J. (1998). Treatment of an unusual case of masochism. J. Sex Marital Ther..

[27] Amelung T., Kuhle L.F., Konrad A., Pauls A., Beier K.M. (2012). Androgen deprivation therapy of self-identifying, help-seeking pedophiles in the Dunkelfeld. Int. J. Law Psychiatry.

[28] Bradford J.M., Pawlak A. (1993). Double-Blind Placebo Crossover Study of Cyproterone Acetate in the Treatment of the Paraphilias. Arch. Sex. Behav..

[29] Boons L., Jeandarme I., Vervaeke G. (2021). Androgen Deprivation Therapy in Pedophilic Disorder: Exploring the Physical, Psychological, and Sexual Effects From a Patient’s Perspective. J. Sex. Med..

[30] Cooper A.J., Cernovsky Z.Z. (1994). Comparison of Cyproterone Acetate (CPA) and Leuprolide Acetate (LHRH Agonist) in a Chronic Pedophile: A Clinical Case Study. Biol. Psychiatry.

[31] Kiersch T.A. (1990). Treatment of Sex Offenders with Depo-Provera. Bull. Am. Acad. Psychiatry Law.

[32] Kravitz H.M., Haywood T.W., Kelly J., Wahlstrom C., Liles S., Cavanaugh J.L. (1995). Medroxyprogesterone Treatment for Paraphiliacs. Bull. Am. Acad. Psychiatry Law.

[33] Krueger R.B., Hembree W., Hill M. (2006). Prescription of medroxyprogesterone acetate to a patient with pedophilia, resulting in Cushing’s Syndrome and adrenal insufficiency. Sex. Abus. J. Res. Treat..

[34] Lehne G.K., Money J. (2000). The First Case of Paraphilia Treated With Depo-Provera: 40-Year Outcome. J. Sex Educ. Ther..

[35] Meyer Ill W.J., Cole C., Emory E. (1992). Depo Provera Treatment for Sex Offending Behavior: An Evaluation of Outcome. Bull. Am. Acad. Psychiatry Law.

[36] Choi J.H., Lee J.W., Lee J.K., Jang S., Yoo M., Lee D.B., Hong J.-W., Noh I.S., Lim M.H. (2018). Therapeutic effects of leuprorelin (leuprolide acetate) in sexual offenders with paraphilia. J. Korean Med. Sci..

[37] Saleh F. (2005). A hypersexual paraphilic patient treated with leuprolide acetate: A single case report. J. Sex Marital Ther..

[38] Dickey R. (1992). The Management of a Case of Treatment-Resistant Paraphilia with a Long-Acting LHRH Agonist. Can. J. Psychiatry.

[39] Habermeyer B., Händel N., Lemoine P., Klarhöfer M., Seifritz E., Dittmann V., Graf M. (2012). LH-RH agonists modulate amygdala response to visual sexual stimulation: A single case fMRI study in pedophilia. Neurocase.

[40] Schiffer B., Gizewski E., Kruger T. (2009). Reduced neuronal responsiveness to visual sexual stimuli in a pedophile treated with a long-acting LH-RH agonist. J. Sex. Med..

[41] Dickey R. (2002). Case report: The management of bone demineralization associated with long-term treatment of multiple paraphilias with long-acting LHRH agonists. J. Sex Marital Ther..

[42] Landgren V., Malki K., Bottai M., Arver S., Rahm C. (2020). Effect of gonadotropin-releasing hormone antagonist on risk of committing child sexual abuse in men with pedophilic disorder: A randomized clinical trial. JAMA Psychiatry.

[43] Landgren V., Olsson P., Briken P., Rahm C. (2022). Effects of testosterone suppression on desire, hypersexuality, and sexual interest in children in men with pedophilic disorder. World J. Biol. Psychiatry.

[44] Rösler A., Witztum E. (1998). Treatment of men with paraphilia with a long-acting analogue of gonadotropin-releasing hormone. N. Engl. J. Med..

[45] Rousseau L., Couture M., Dupont A., Labrie F., Couture N. (1990). Effect of combined androgen blockade with an LHRH agonist and flutamide in one severe case of male exhibitionism. Can. J. Psychiatry.

[46] Shiah I.-S., Chao C.-Y., Mao W.-C., Chuang Y.-J. (2006). Treatment of paraphilic sexual disorder: The use of topiramate in fetishism. Int. Clin. Psychopharmacol..

[47] Vayısoğlu S. (2023). Symptoms of exhibitionism that regress with bupropion: A case report. Front. Psychiatry.

[48] Pearson H.J., Marshall W.L., Barbaree H.E., Southmayd S. (1992). Treatment of a Compulsive Paraphiliac with Buspirone. Ann. Sex Res..

[49] Overall J.E., Gorham D.R. (1962). The Brief Psychiatric Rating Scale.

[50] Thibaut F., Cosyns P., Fedoroff J.P., Briken P., Goethals K., Bradford J.M.W. (2020). The World Federation of Societies of Biological Psychiatry (WFSBP) 2020 guidelines for the pharmacological treatment of paraphilic disorders. World J. Biol. Psychiatry.

[51] Bickle A., Cameron C., Hassan T., Safdar H., Khalifa N. (2021). International overview of phallometric testing for sexual offending behaviour and sexual risk. BJPsych Int..

[52] Marshall W.L. (2014). Phallometric assessments of sexual interests: An update. Curr. Psychiatry Rep..

[53] Kolářský A., Freund K., Machek J., Polak O. (1967). Male sexual deviation: Association with early temporal lobe damage. Arch. Gen. Psychiatry.

